# Storage Stability of Nutritional Qualities, Enzyme Activities, and Volatile Compounds of “Hangjiao No. 2” Chili Pepper Treated With Different Concentrations of 1-Methyl Cyclopropene

**DOI:** 10.3389/fpls.2022.838916

**Published:** 2022-03-08

**Authors:** Emily Patience Bakpa, Jing Zhang, Jianming Xie, Yufeng Ma, Kangning Han, Youlin Chang

**Affiliations:** Gansu Agricultural University, College of Horticulture, Lanzhou, China

**Keywords:** aroma, “Hangjiao No.2” chili pepper, 1-MCP, quality, antioxidant enzyme activities

## Abstract

This study aimed to determine the effects of different concentrations of 1-methyl cyclopropene (1-MCP) on the nutritional quality, antioxidant enzyme activities, and volatile compounds of “Hangjiao No.2” chili pepper during 12 days of storage at ambient temperature. The chili fruit were randomly selected and divided into four groups corresponding to the four treatments, thus, 0.5, 1.0, and 1.5 μl L^–1^ 1-MCP and a control. The analysis of the nutritional value, enzyme activities, and volatile compounds were determined at 3 days interval. The results showed that the malondialdehyde (MDA) content was lower in the fruit treated with 1-MCP compared to the control. The treatment with 1.5 μl L^–1^ and the control showed the lowest superoxide dismutase (SOD) activity compared to the other treatments. Peroxidase (POD) and Catalase (CAT) were highest in the fruit treated with 0.5 μl L^–1^ compared to the control and treatment with 1.0 μl L^–1^. The 1.5 μl L^–1^ treatment delayed the decline in vitamin C and protein content compared to the control. Nitrate levels increased 1.34-fold at 0.5 μl L^–1^ and 2.01-fold in the control. Chlorophyll content degradation was delayed at 1.0 μl L^–1^ compared to the control. A total of 88 volatile compounds, including terpenes, aldehydes, alkanes, esters, alcohols, acids, phenolic derivatives, ketones, and other aromatic compounds, were detected in “Hangjiao No.2” pepper during the 12-day storage period and treatment concentrations. The production of volatile terpenes was higher in the control than in the 1-MCP treatments, while the 0.5 μl L^–1^ 1-MCP treatment generally suppressed the production of volatile compounds during storage. Overall, the production of volatile compounds after treatment was higher in the “Hangjiao No.2” chili fruit treated with 1.0 μl L^–1^ 1-MCP than in the other treatments throughout the storage period. The results indicate that 1-MCP treatment was more effective in maintaining fruit quality, enhancing the activities of SOD, POD, and CAT, retarding the accumulation of MDA and restoring volatile aromas, with 1.0 μl L^–1^ having the best preservative effect on “Hangjiao No.2” chili fruit during storage, which could be useful for future marketing and processing.

## Introduction

Chilis, botanically known as *Capsicum annuum* L., belongs to the Solanaceae family and is considered one of the most widely cultivated crops used as an all-purpose spice (Batiha et al., 2020). The fruits are rich in vitamins such as vitamins A, C, E, and B6, beta-carotene, folic acid, and thiamine and are mainly cultivated for their greens, sauces, prickles, and special taste and aroma (Bosland and Votava, 2012). The aldehydes and volatile terpenes of pepper have also recently attracted the attention of many researchers and the food industry as they are excellent antimicrobial additives with very low residues for food preservation (Mercy, 2018). Dias (2012) stated that the capsaicin extracted from chili peppers increases heat production and oxygen consumption in the body, which promotes calorie burning and weight loss. In addition, the chili pepper plant provides an effective and inexpensive source with great economic and potential health benefits for controlling the development of various metabolic and antioxidant-associated diseases in contrast to treatments with synthetic drugs, which are not only expensive but also have side effects and become increasingly resistant with prolonged use (Mercy, 2018). Commercially, the fruit is of great importance as it is the most important element in a variety of cuisines around the world, adding flavor, aroma, and color to dishes (Chandra Sekhar Rao, 2014). The annual cultivated area of pepper in China is more than 13,000 km^2^, which makes pepper one of the most widely grown vegetables (Ou et al., 2017).

“Hangjiao No.2” is a widely grown chili pepper cultivated in northern China, and its sources of various nutrients are crucial for dietary diversification and national food security. The plant is usually harvested and consumed at the green, mature stage. The fruits of pepper plants vary in pungency due to the presence of capsaicinoids in varying concentrations (Asnin and Park, 2015) and are mainly used for seasoning many Chinese cuisines. However, the market value of pepper fruits is limited by post-harvest conditions due to their short shelf-life. The shelf-life of pepper is within 3–5 weeks, especially when stored in moisture-retaining films at 7.5°C, and less than 2 weeks when stored at 5°C (Fernández-Trujillo et al., 2009). However, neither the distribution chain nor consumers usually have the ability to store a particular product under such ideal conditions, so compromises must be made in the choice of temperature and relative humidity. Due to the increasing consumer demand for products of superior quality and high nutritional content, the optimization of postharvest conditions to preserve the natural green color and quality of the pepper fruit is the focus of this study.

1-Methylcyclopropene (1-MCP) is a cyclic alkene and also an ethylene antagonist that binds to ethylene receptors in plant cells and inhibits ethylene binding, preventing signal transduction and ethylene action (Lurie, 2006; Serek et al., 2015). 1-MCP is one of the postharvest agents used to delay spoilage, thus improving the shelf-life, quality, and flavor of many climacteric fruit and vegetables. However, it was found that 1-MCP can also show a significant preservative effect in inhibiting the development of physiological disorders, discoloration, senescence, and color change of some non-climacteric fruit and vegetables such as broccoli, eggplant, pitaya, and jujube (Massolo et al., 2011; Zhang et al., 2012b; Fernández-León et al., 2013; Li et al., 2016). According to studies by Chae et al. (2009), fruits treated with 1-MCP had significantly lower lipoxygenase activities and electrolyte losses during storage than the control group, showing the potential benefits of 1-MCP. The effect of 1-MCP on storage disorders depends on the cultivar, the presence or absence of ethylene, and the concentration used (Salvador et al., 2005). Many studies have shown that 1-MCP protects agricultural products, especially fruits, from exogenous and self-generated ethylene, extends postharvest shelf-life, and provides more flexibility in storage, distribution, and sale (Watkins and Miller, 2005; Watkins, 2008). 1-MCP is non-toxic, leaves little residue, and is active at very low concentrations. Due to its availability and stable complexed formulation that releases 1-MCP when dissolved in water, it has attracted research and commercial interest worldwide (Watkins and Miller, 2005). Although there are several reports in the literature on the response of horticultural crops to different concentrations of 1-MCP (Zhang et al., 2012a,^2020^; Jiang et al., 2018; Hussain et al., 2019; Li et al., 2021), the available information on the effects of 1-MCP on the changes that occur in volatile aroma compounds, antioxidant enzyme activities, and internal qualities of peppers during storage is scanty. Considering that aroma, color, texture, and nutritional properties are the most important factors affecting the overall quality of pepper fruits, this study aimed to evaluate the mechanisms of different concentrations of 1-MCP on the stability of nutritional values, antioxidant enzyme activities, and volatile compounds of “Hangjiao No.2” pepper during storage.

## Materials and Methods

### Pepper

“Hangjiao No.2” pepper is a high-yielding chili hybrid developed by Tianshui Shenzhou Lvpeng Agricultural Technology Co., Ltd., and is widely grown in northwestern China. The pepper exhibits a climacteric behavior as evidenced by a preliminary test that was conducted on it (Supplementary Material). The “Hangjiao No.2” peppers were harvested from pepper plants treated with 1.8 kg of amino acid water-soluble fertilizer (organic fertilizer) containing aspartic acid (10.62 g L^–1^), threonine (4.75 g L^–1^), serine (17.38 g L^–1^), glutamic acid (9.91 g L^–1^), glycine (19.39 g L^–1^), valine (7.39 g L^–1^), methionine (0.65 g L^–1^), isoleucine (4.67 g L^–1^), leucine (3.87 g L^–1^), tyrosine (1.15 g L^–1^), phenylalanine (6.81 g L^–1^), lysine (2.61 g L^–1^), arginine (9.06 g L^–1^), and proline (13.92 g L^–1^) in Lintao County, Gansu Province, China. Mature pepper fruit were packed in plastic boxes according to treatment and transported to the laboratory after harvest. Pepper fruit with uniform size and no diseases were randomly selected, divided into groups, and packed into perforated 40 L plastic containers.

### Application of 1-MCP Treatments

The “Hangjiao No.2” chili fruit were randomly selected after harvest and divided into four groups. Each group consisted of 120 fruit with three replicates. Each group was placed in a 40 L plastic container and treated with 0.5, 1.0, and 1.5 μl L^–1^ 1-MCP. The containers were sealed with a 4.98 mm thick plastic film for 12 h at an ambient temperature, while the non-treated group served as a control. All experimental groups were stored immediately after treatment at an ambient temperature of 22 ± 1°C and 75 ± 50% relative humidity (RH). Pepper samples (5) were used for the evaluation of indices at 3-day intervals for 12 days.

### Determination of Chlorophyll Content

The chlorophyll content was determined according to the method described by Lichtenthaler (1987). Randomly selected pepper samples at 3 days intervals from each treatment were weighed (1.0 g) and ground in a mortar. In total, 5 ml of acetone (80%) was added to the mixture to obtain a homogenate. Added to the mixture was 10 ml of the same acetone until it turned white. The homogenate was filtered with filter paper into a 50 ml volumetric flask after allowing the mixture to stand for 6 min. Acetone (80%) was again added to the filtrate until the final mark was reached on the 25 ml flask. Finally, the supernatant was used for colorimetric determination at 440, 645, and 663 nm colorimetric wavelengths with five independent replicates.

### Measurement of Vitamin C, Nitrates, and Protein Contents

Five mature fruit from each treatment were randomly selected at 3 days intervals during storage with five independent replicates for internal quality parameters. Vitamin C was measured using the 2,6-dichloroindophenol staining method described by Arya et al. (2000). The salicylic acid method was used to quantify nitrate content (Cataldo et al., 1975). Protein content was determined by the Coomassie Brilliant Blue method (Sedmak and Grossberg, 1977). The results are expressed in mg g^–1^ on a fresh weight basis.

### Evaluation of Antioxidant Enzyme Activities of Superoxide Dismutase, Peroxidase, Catalase, and Malondialdehyde Content

The superoxide dismutase (SOD) activity was measured by weighing 1.0 g of each pepper sample using an electronic balance. The weighed samples were then ground in a mortar and pestle on ice with 5 ml of the extraction solution in 50 mM sodium phosphate (pH 7.8). The reaction buffer (5 ml) consisted of 50 MM sodium phosphate buffer (pH 7.8), 130 MM methionine, nitroblue tetrazolium (750 μm), EDTA-Na_2_ (100 μM), riboflavin (20 μM), and enzyme extract (0.1 ml). After shaking, each mixture was irradiated with light (60 mol m^–2^ s^–1^) for 20 min and absorbance was then measured at 560 nm. Each solution was repeated three times. The activity of SOD was expressed as μg^–1^ on a fresh weight basis, where the unit indicates the amount of enzyme that causes a 50% decrease in the reduction of SOD habitable nitroblue tetrazolium per mass of pepper fruit per hour, a procedure described by Abassi et al. (1998). The method of Reuveni (1992) was used to estimate the activity of peroxidase (POD). Weighed pepper samples (1.0 g) were ground in a mortar and pestle on ice with 5 ml of the extraction solution containing polyvinylpolypyrrolidone (PVPP) (4%), Triton (x-100), and polyethylene glycol (PEG) (1 mmol). The homogenates were centrifuged at 12,000 *g* for 30 min at 4°C. After centrifugation, the supernatants were then used to measure the enzymatic activities. A crude enzyme extraction buffer containing 3 ml of guaiacol (25 mM) was used as substrate. The extraction buffer (30 μl) was mixed with 50 μl of H_2_0_2_ (30%) and the mixture was then transferred to a cuvette in the spectrophotometer chamber along with distilled water as a reference for POD evaluation. The absorbance values were then read at 470 nm per 20 s. The BEERS and SIZER’s (1952) method was used to evaluate the catalase (CAT) activity. The reaction mixture contained enzyme extract (0.1 ml), phosphate buffer (50 mM) with pH 7.0, and hydrogen peroxide (15 mM). The degradation of hydrogen peroxide was determined by measuring the change in absorbance at 240 nm using a spectrophotometer. One unit of CAT activity was defined as the amount of enzyme required to reduce 1μmol of H_2_0_2_ in 1 min. The malondialdehyde (MDA) content of pepper fruit for the different treatments was determined using the thiobarbituric acid reaction method, a procedure described by Li et al. (2015). A weighed sample (0.3 g) from each treatment was homogenized in 3 ml of 0.05 mol acid buffer (pH 7.8). Thiobarbituric acid (5 ml) at a concentration of 0.5% was added to the solution and mixed thoroughly. The mixture was then transferred to a 10 ml test tube and placed in a boiling water bath for 10 min. The test tube containing the solution was then cooled after 10 min in the water bath. The cooled test tube was then centrifuged at 3,000 *g* for 15 min. The supernatant was taken and the absorbance was determined at 532, 600, and 450 nm with 0.5% barbituric acid solution of the sixth generation. The results are expressed in μmol g^–1^ on a fresh weight basis.

### Evaluation of the Volatile Compounds

The volatile compounds were prepared by grinding 8 g of pepper fruit from each treatment with a small amount of silica sand in a mortar and pestle. The ground fruit were then transferred to a 20 ml vial. Sodium chloride (1 g) and 82.1 μg L^–1^ 2-octanol (10 μl) were added to the vial containing the ground sample along with a small magnetic stirrer. The vial was then tightly sealed and equilibrated in a 40°C water bath at 40 rmp agitation for 30 min. The volatile compounds in the headspace of the vial were absorbed by a 50/30 μm divinyl benzene/carboxen/polydimethylsiloxane (DVB/CAR/PDMS) fiber. The fiber was thermally desorbed into the gas chromatography/mass spectrometer (GC-MS) injector port for approximately 10 min after extraction, with each sample repeated three times. Volatile compounds were determined by gas chromatography-mass spectrometry (TRACE:1310-ISQ, Thermo Fisher Scientific San Jose Clara, CA, United States). Helium with an fbw of 1 ml min^–1^ was used as the carrier gas. The gas chromatography (GC) temperature started at 50°C for 10 min, followed by a temperature series of 3°C min^–1^ to the final temperature of 180°C and 6 min final time. The ion source temperature was 250°C, while the transfer line temperature was 180°C. The mass ranged from 50 to 350 m/z with an electron energy of 70 eV in full scan mode. The various detected aromatic compounds were measured by comparing their mass spectra with those of the National Institute for Standard and Technology library (NIST14 version 2). The retention indices were measured using the C6-C21 n-alkane series (supelco, Bellefonte, PA, United States) and compared with the values reported in the literature. The compounds were quantitatively evaluated based on 2-octanol (internal standard) according to a method described by Wu et al. (2020) and Yuan and Qian (2016) with a slight modification.

### Statistical Analysis

Statistical analysis was carried out using the SPSS package program (version 20.0 SPSS Institute Ltd., United States). The data collected at each time point were analyzed using a one-way ANOVA model, the variants being storage time and 1-MCP treatments. Means were tested for significant differences at *p* < 0.05 using Tukey’s Honest. All data were expressed as means ± SEs.

## Results

### Changes in MDA A, SOD B, POD C, and CAT D of “Hangjiao No.2” Chili Pepper Treated With Different Concentrations of 1-MCP and the Control During 12 Days Storage at Ambient Temperature

**FIGURE 1:**
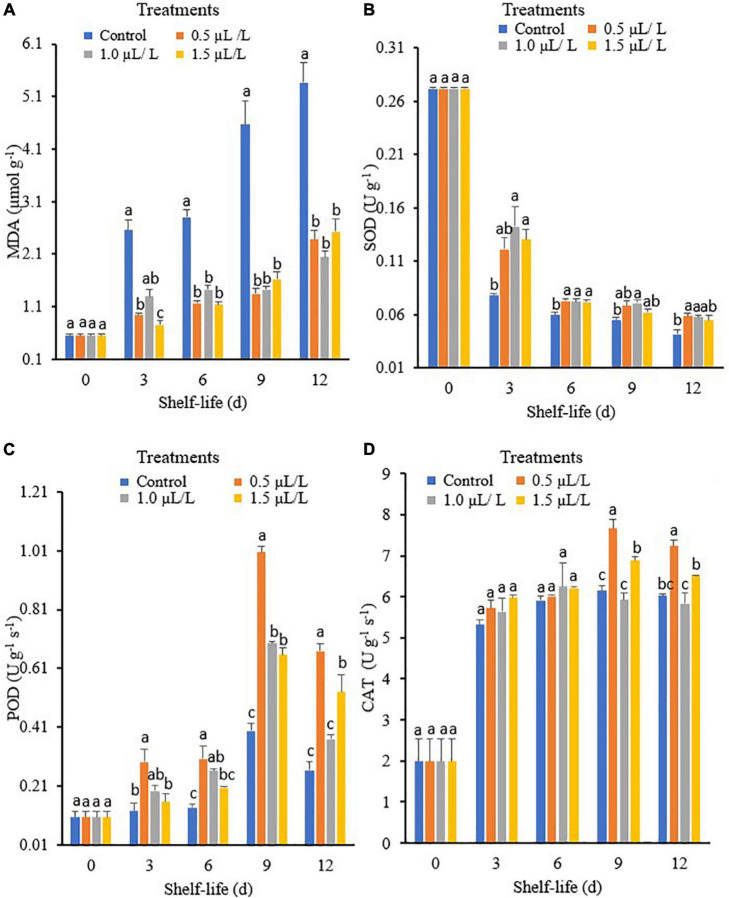
Malondialdehyde (MDA) **(A)**, superoxide dismutase (SOD) **(B)**, peroxidase (POD) **(C)**, and catalase (CAT) **(D)** of green mature “Hangjiao No.2” chili peppers treated with different concentrations of 1-methyl cyclopropane (1-MCP) and the control during 12 days storage at ambient temperature. Each value is the mean of five replicates and each bar represents ± standard error.

Malondialdehyde (MDA) is one of the most important indicators of membrane lipid peroxidation, which indicates cell membrane integrity. This enzyme is also involved in the synthesis of antioxidants. Figure 1A shows that the MDA content varied considerably with the different storage times and treatment concentrations. The MDA content was higher in all treated fruit as the storage period progressed, although the MDA content was lower in the fruit treated with 1-MCP than in the control. In addition, the MDA content of the pepper fruit in the control was higher than that of the fruit treated with 1.5 μl L^–1^ 1-MCP on day 3. However, there were no significant differences in MDA levels between the 1-MCP treatments at days 6, 9, and 12, which were lower compared to the control.

Superoxide dismutase (SOD) is an important enzyme associated with fruit and vegetable senescence and defense responses that protect cells from oxidative damage by scavenging reactive oxygen species (Lee and Lee, 2000). The activity of SOD varied significantly under different storage times and treatments as shown in Figure 1B. On day 3, the highest SOD activity was observed when the fruit was treated with 1-MCP at 1.0 μl L^–1^, followed by 1.5 and 0.5 μl L^–1^, while the control had the lowest SOD activity. When stored at 6 days, there were no significant differences in the activity of SOD at the treatment concentrations of 0.5, 1, and 1.5 μl L^–1^ and they were all higher than the control. The highest SOD activity on day 9 was observed in the 1-MCP at 1.0 μl L^–1^, while the control had the lowest activity. However, there were no significant differences in SOD activities between the control and the 1.5 μl L^–1^ treatment and the control, which had the lowest SOD activity on day 12.

The POD activity varied significantly among the different storage times and treatments, as shown in Figure 1C. On day 3, the highest enzyme activity was observed in the 0.5 μl L^–1^ treatment, which was insignificant in the 1.0 μl L^–1^ treatment compared to the 1.5 μl L^–1^ treatment or control. The 0.5 and 1.5 μl L^–1^ treatments exhibited the highest POD activity on day 6 of storage compared to the control. Moreover, the activity of POD increased and peaked on day 9 and then decreased at the end of storage, with the 0.5 μl L^–1^ treatment showing the highest activity, while the control and the 1.0 μl L^–1^ treatment showed the lowest activity at day 12. The obtained results indicate that treatment with 1-MCP can induce the activities of POD, and 0.5 μl L^–1^ increased the POD activity of pepper the most.

The CAT activity showed an increasing pattern at 3, 6, and 9 days in all treatments and then began to decline during the storage period (Figure 1D). However, pepper fruit treated with 0.5 μl L^–1^ showed the highest CAT activity after 6 days of storage compared to control and 1.0 μl L^–1^ treatment at 9 and 12 days of storage, respectively.

### Changes in Vitamin C, Nitrate, Protein, and Chlorophyll Content of Green Mature “Hangjiao No.2” Pepper Treated With Different Concentrations of 1-MCP During Storage at Ambient Temperature

Vitamin C is an important antioxidant of pepper and neutralizes superoxide and hydroxyl radicals due to its water solubility (Podsedek, 2007). According to Tan et al. (2012), factors such as harvesting condition, storage duration, and environmental conditions have an influence on the constituents of harvested vegetables. Vitamin C content decreased in all treatments with advancing storage period in our study. However, 1-MCP at a concentration of 1.5 μl L^–1^ retarded the decline of vitamin C in pepper fruit (0.46 mg g^–1^) during storage period compared to the control (0.36 mg g^–1^), 0.5 μl L^–1^ treatment (0.40 mg g^–1^), and 1.0 μl L^–1^ treatment (0.41 mg g^–1^) as presented in Table 1.

**TABLE 1:** Changes in the vitamin C, nitrate, protein, and chlorophyll content of green mature “Hangjiao No.2” peppers treated with different concentrations of 1-MCP during storage at ambient temperature.

Elements	Storage period (d)	Treatments			
		Control	0.5 μl L^–1^	1 μl L^–1^	1.5 μl L^–1^
Vitamin C (mg g^–1^)	0	2.56 ± 0.05	2.56 ± 0.05	2.56 ± 0.05	2.56 ± 0.05
	3	0.54 ± 0.01b	0.53 ± 0.02b	0.71 ± 0.02a	0.77 ± 0.02a
	6	0.51 ± 0.03b	0.52 ± 0.02b	0.66 ± 0.03a	0.72 ± 0.02a
	9	0.44 ± 0.02b	0.50 ± 0.03ab	0.49 ± 0.02ab	0.52 ± 0.02a
	12	0.36 ± 0.01b	0.40 ± 0.01b	0.41 ± 0.01b	0.46 ± 0.02a
Nitrate (mg g^–1^)	0	3.95 ± 0.15	3.95 ± 0.15	3.95 ± 0.15	3.95 ± 0.15
	3	5.15 ± 0.07b	4.77 ± 0.12bc	4.36 ± 0.13c	5.70 ± 0.16a
	6	6.55 ± 0.07a	4.95 ± 0.06c	5.18 ± 0.03c	6.14 ± 0.13b
	9	7.34 ± 0.18a	5.16 ± 0.05b	5.54 ± 0.08b	6.91 ± 0.06a
	12	7.95 ± 0.07a	5.31 ± 0.07c	6.23 ± 0.18b	6.96 ± 0.04b
Protein (mg g^–1^)	0	3.39 ± 0.17	3.39 ± 0.17	3.39 ± 0.17	3.39 ± 0.17
	3	2.01 ± 0.06c	2.32 ± 0.08b	2.44 ± 0.04b	2.72 ± 0.03a
	6	1.88 ± 0.06c	2.08 ± 0.04b	2.00 ± 0.04bc	2.46 ± 0.02a
	9	1.62 ± 0.03c	1.97 ± 0.03b	1.93 ± 0.03b	2.35 ± 0.05a
	12	1.57 ± 0.02c	1.70 ± 0.02c	1.90 ± 0.04b	2.23 ± 0.05a
Chlorophyll (mg g^–1^)	0	0.48 ± 0.00	0.48 ± 0.00	0.48 ± 0.00	0.48 ± 0.00
	3	0.33 ± 0.00c	0.31 ± 0.00d	0.43 ± 0.00a	0.42 ± 0.00b
	6	0.19 ± 0.01b	0.18 ± 0.02b	0.23 ± 0.01a	0.20 ± 0.01ab
	9	0.06 ± 0.00a	0.06 ± 0.00a	0.07 ± 0.00a	0.05 ± 0.00a
	12	0.03 ± 0.00a	0.03 ± 0.00a	0.05 ± 0.00a	0.04 ± 0.00a

*Data expressed as means ± standard errors of five measurements. Different subscripts within the same column indicate significant differences (Turkey’s test p < 0.05).*

Consumption of vegetables promotes diseases such as blue baby syndrome and cancer when the conversion of nitrate to nitrides occurs in various processes in the human body (Salehzadeh et al., 2020). The nitrate content of vegetables varies due to storability, harvesting time, cultivar, traditional, and greenhouse methods, temperature, moisture stress, light intensity, and storage conditions (Colla et al., 2018). The nitrate levels determined in this study were significantly increased in the control compared to the 1-MCP treated fruit (Table 1). Moreover, the 0.5 μl L^–1^ under the 1-MCP treatments increased the nitrate content by 1.34-fold compared to the control, which increased the nitrate content by 2.01-fold during the 12 days storage.

Protein content decreased among the different treatments in this study as the storage period progressed, but the 1-MCP treatments inhibited the decrease in protein content compared to the control. Among the 1-MCP treatments, 1.5 μl L^–1^ reduced protein content by 34.22% during the 12-day storage period, while the control was reduced by 53.69% (Table 1).

All the treatments applied in this study caused a significant decrease in the total chlorophyll content of the pepper fruit during 12 days of storage (Table 1). Although the reduction in pepper fruit treated with 1.0 μl L^–1^ was minimized under the 1-MCP concentrations compared to the control on days 3 and 6, the differences between all treatments on days 9 and 12 were not significant. 1-MCP has been reported to delay chlorophyll degradation in broccoli florets (Fernández-León et al., 2013), *Solanum melongena*, L (Massolo et al., 2011), Yardlong bean (Jiang et al., 2018), and strawberries (Li et al., 2016). This could be the reason for the observed delay in chlorophyll content in pepper fruit treated with 1.0 μl L^–1^ during the storage period.

### Changes in the Volatile Aroma Substances of the “Hangjiao No.2” Chili Pepper

**FIGURE 2:**
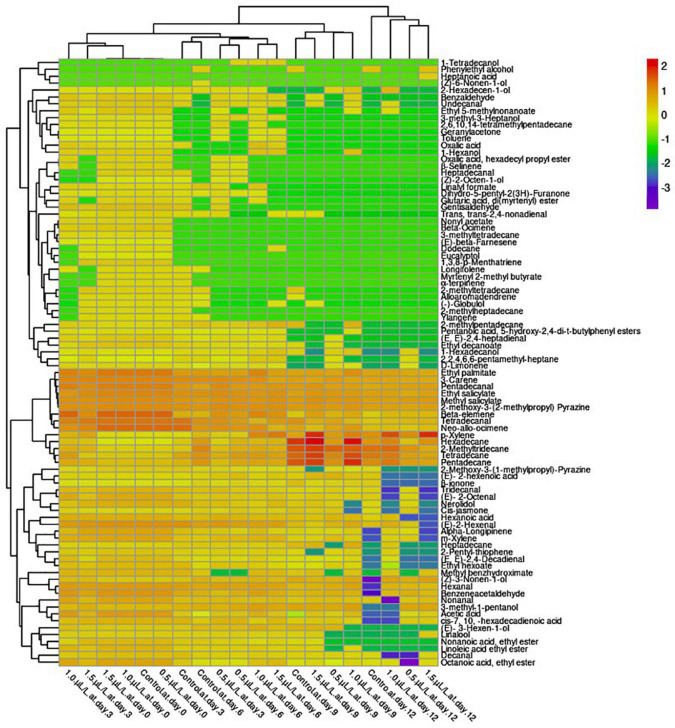
Hierarchical clustering of a heat map showing the changes in the volatile compounds of “Hangjiao No.2” chili peppers under the influence of different concentrations of 1-MCP (0.5, 1.0, and 1.5 μl L^–1^) and control during 12 days of storage period. The different treatments were shown in columns. Volatile compounds were plotted in rows. The color box indicates the abundance of each compound.

Aroma is another important component that influences product satisfaction. However, the perceived aroma is a complex mixture of several volatile compounds (Schiller et al., 2015). As shown in the heat map, a total of 88 volatile compounds were detected, which were classified into different classes such as acids, aldehydes, alkanes, esters, terpenes, alcohols, ketones, and others based on the hierarchical clustering (Figure 2). The concentration of the classes of volatile compounds varied greatly between treatments and storage time. The profiles of fruit volatile acids include acetic acid, oxalic acid, hexanoic acid, (E)-2-hexenoic acid, cis-7, 10, -hexadecadienoic acid, heptanoic acid, and glutaric acid, di(myrtenyl) ester. The results show that pepper fruit treated with 1.0 μl L^–1^ 1-MCP were the most affected and had the highest content of acids with increasing storage time compared to 0.5 μl L^–1^, 1.5 μl L^–1^, and the control, respectively. The content of hexanoic acid decreased with increasing storage time in all treatments, with the treatment having the highest value at 1.0 μl L^–1^. The content of (E)-2-hexenoic acid in the pepper fruit increased with increasing storage period. However, the content of (E)-2-hexenoic acid was not detected in the fruit treated with 1-MCP at the end of storage, except in the control. The treatments with 1.0 μl L^–1^ resulted in the highest postharvest acetic acid contents on days 3, 6, and 9 compared to the other treatments. The 0.5 μl L^–1^ 1-MCP treatment increased cis-7, 10, -hexadecadienoic acid content at days 3, 6, 9, and 12 compared to the control and the other 1-MCP treatments. No value was detected in the 1.0 μl L^–1^ treatment and the control on day 12. Glutaric acid, di(myrtenyl) ester content was detected only on day 3 in the control and 0.5 μl L^–1^, respectively, but disappeared thereafter, while fruit treated with 1.0 μl L^–1^ were relatively stable and diminished on day 6 during storage. Oxalic acid was detected only on days 3 and 6 for the control and the fruit treated with 1.5 and 1.0 μl L^–1^, respectively, with 1.0 μl L^–1^ showing the highest content. Heptanoic acid, on the other hand, was detected only at the end of storage for the 1.5 μl L^–1^ treatment.

Qualitatively, 16 aldehydes were identified in pepper fruit and the results showed that all aldehydes had the tendency to decrease or diminish under all treatments applied during the 12-day storage period. The content of hexanal decreased in all treated fruit during the 12-day storage period, with 1.0 μl L^–1^ treatment having the highest content compared to the control, which diminished at the end of storage. The levels of pentadecanal and tetradecanal in the pepper fruit showed a decreasing trend in all treatments as the storage period progressed and were quite stable at day 3, but decreased rapidly, with the 0.5 and 1.5 μlL^–1^ treatments showing the highest level at the end of storage compared to the control. During storage, the (E, E)-2,4-heptadienal content of pepper fruit decreased in the treatments with 1.0 and 1.5 μl L^–1^ after 6 days, except for the treatment with 0.5 μl L^–1^, which decreased after 9 days. The highest content of benzeneacetaldehyde was obtained in the 0.5 μl L^–1^ treated pepper fruit at the end of storage, while no value was detected in the control-treated fruit. On the other hand, nonanal content also decreased with increasing storage time. As can be seen in Figure 2, the 1.5 μl L^–1^ 1- MCP treatment delayed the reduction of nonanal compared to the control. Tridecanal disappeared at the end of storage in fruit treated with 1.0 and 1.5 1-MCP compared to the control, which had the highest level. The (E)-2-hexenal in all treated pepper fruit dropped sharply during storage. However, the 1.0 μl L^–1^ treatment further delayed the decline of (E)-2-hexenal in the fruit compared to the other treatments but inhibited the concentration of (E)- 2-octenal at the end of storage along with the 0.5 μl L^–1^ treatment. The content of gentisaldehyde was detected in all treated fruit only on day 3 of storage after the initial stage, with the highest gentisaldehyde content found in the 1.0 μl L^–1^ treated pepper fruits. Heptadecanal was detected before day 6 in fruit treated with 0.5 μl L^–1^ and benzaldehyde before day 9 in both fruit treated with 1.0 and 1.5 μl L^–1^ compared to the control.

In this study, alkanes such as tetradecane, pentadecane, hexadecane, 2-methyltridecane, heptadecane, 2,2,4,6,6-pentamethyl-heptane, 2-methylpentadecane, 2,6,10,14-tetramethylpentadecane, 3-methyltetradecane, 2-methyltetradecane, 2-methylheptadecane, and dodecane were identified. The levels of tetradecane, pentadecane, hexadecane, 2-methyltridecane and heptadecane increased with increasing storage time, reached a maximum on day 9 and then decreased on day 12 in all treatments, with the control having the highest tetradecane, while 1.0 μl L^–1^ had the highest levels of pentadecane, hexadecane, 2-methyltridecane, and heptadecane at the end of storage. The concentrations of decanal, trans, trans-2,4-non-adienal, undecanal, and dodecane of “Hangjiao No.2” were produced in trace elements. Moreover, decanal and undecanal were detected only after 6 and 12 days in the control and 1.5 μl L^–1^ treatments, respectively, and maintained the trans, trans-2,4-non-adienal of the fruit until the 9th day, while 1-MCP at 1.0 μl L^–1^ inhibited the disappearance of dodecane during 12 days of storage. The 2,2,4,6,6-pentamethyl-heptane increased before day 6 and decreased on day 9 in the control and in the peppers treated with 1.0 and 1.5 μl L^–1^, while the 2,2,4,6,6-pentamethyl-heptane in the peppers treated with 0.5 μl L^–1^ continued to increase at a lower level until the end of storage. Concentrations of 2-methylpentadecane and 2-methyltetradecane also increased during storage but began to diminish after 6 days in the samples treated with 0.5 and 1.5 μl L^–1^ and after 9 days in the control (Figure 2). The 1-MCP treatments increased the levels of 2,6,10,14-tetramethylpentadecane and 3-methyltetradecane in the fruit before day 3 and then decreased after day 6 of storage in the 0.5 and 1.5 μl L^–1^ treatments and 1.0 μl L^–1^ on day 9. Moreover, the 0.5 and 1.0 μl L^–1^ treatments inhibited the production of 2-methylheptadecane after the initial stage during storage compared to the control and the 1.5 0 μl L^–1^ treatment.

According to Thewes et al. (2015), esters are the main volatile compounds produced by apples. Twelve esters such as ethyl salicylate, linoleic acid ethyl ester, octanoic acid, ethyl ester, myrtenyl 2-methyl butyrate, ethyl palmitate, ethyl decanoate, nonanoic acid, ethyl ester, ethyl hexoate, oxalic acid hexadecyl propyl ester, pentanoic acid 5-hydroxy- 2,4-di-t-butylphenyl, ethyl 5-methylnonanoate, and nonyl acetate were found in “Hangjiao No.2” chili fruit in this study after harvest, with ethyl salicylate being the most abundant. All the detected esters have the tendency to decrease or diminish during storage (Figure 2). In summary, 1.0 μl L^–1^ under the 1-MCP treatments retained most of the desired esters better, especially before day 6 during the storage period compared to the control.

Terpenes were more dominant in the “Hangjiao No.2” chili fruit than other organic compounds, which were present in trace amounts. Among the 19 terpenes found in the chili fruit after harvest, 3-carene was the most abundant followed by neo-allo-ocimene, beta elemene, nerolidol, 1,3,8-p-Menthatriene, and alpha-Longipinene while d-limonene, alloaromadendrene, longifolene, alpha-longipinene, β-selinene, linalool, €-beta-farnesene, eucalyptol, (−)-globulol, ylangene, α-terpinene, geranylacetone, and β-ionone were in trace amounts. Although the terpene content decreased or disappeared as the storage period progressed, the changes in the terpene content of the “Hangjiao No.2” pepper samples during storage compared with the control pepper samples were less, especially before day 9, than those of the fruit treated with 1-MCP (Figure 2).

Thirteen alcohols were found in this study, accounting for 19% of the total volatiles. Only six of these volatile compounds appeared before day 9, two at the end of storage, and five diminished after day 6, with treatment 1.0 μl L^–1^ showing the highest levels and treatment 0.5 μl L^–1^ inhibiting most alcohols as the day of storage progressed. The most abundant alcohols in the “Hangjiao No.2” chili fruit were 1-hexanol and 3-methyl-1-pentanol, which showed higher levels in the 1.0 μl L^–1^ treatment than in the other treatments during storage. (Z)-6-nonen-1-ol was formed only in control-treated fruit on day 6 of storage, while 1-tetradecanol was formed only in fruit treated with 1-MCP.

Ketones are a type of aroma active compounds that are very important. The composition of cis-jasmone, the only ketone detected in the pepper samples, increased before the 9th day of storage and then decreased at the end of storage in the 0.5 μl L^–1^ treatment and the control, in contrast to the 1.0 and 1.5 μl L^–1^ treatments. The predominant volatile compound detected in the peppers was methyl salicylate, a phenolic derivative (Figure 2). However, its concentration decreased during the storage period, with the highest value being 1.5 μl L^–1^ higher in the treatment than in the control. The control also inhibited the concentration of toluene before day 3 of storage, while the fruit treated with 1-MCP did not. Nevertheless, p-xylene concentrations in the 1.5 μl L^–1^ pepper samples increased significantly during the 12 days storage period compared to the control samples.

Other volatile compounds such as methyl benzhydroximate, 2-pentyl-thiophene, dihydro-5-pentyl-2(3H)-Furanone, 2-methoxy-3-(2-methylpropyl) pyrazine, 1,3-dimethyl-benzene, and 2-methoxy-3-(1-methylpropyl) pyrazine were also detected in “Hangjiao No.2” pepper fruit, and their levels decreased or disappeared during storage (Figure 2). Methyl benzhydroximate showed the highest content, followed by 2-methoxy-3-(2-methylpropyl) pyrazine. However, only 2-methoxy-3-(2-methylpropyl) pyrazine was relatively stable throughout the storage period, although its concentration decreased with storage time in all treatments, with the highest content found in fruit treated with 1.0 μl L^–1^.

**FIGURE 3:**
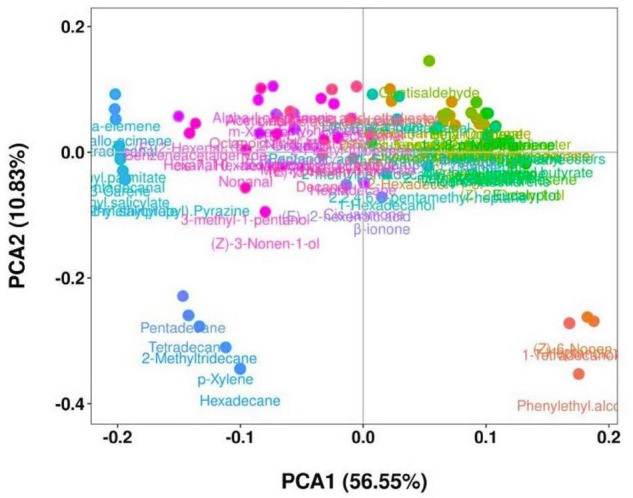
Principal component analysis of volatile compounds of control and 1-MCP treated “Hangjiao No.2” chili peppers at different storage times at ambient temperature.

The evaluation of the volatile compounds that could contribute to the aroma at different storage times of the pepper samples is shown in Figure 3. Multivariate analysis of the results was performed to explain the overall characteristics of the stages based on a reduced number of volatile compounds. The PCA1 and PCA2 were a measure of the green stage pepper samples during 12 days storage period (*p* < 0.05). Figure 3 shows that the major component accounted for 67.38% of the total variability of volatile compounds in the “Hangjiao No.2” chili pepper fruit, indicating a significant difference among treatments. Each treatment corresponded to the stages of storage time (0, 3, 6, 9, and 12 days) separated by PCA1 and PCA2.

## Discussion

Capsicum (*Capsicum annuum*) is a non-climacteric fruit (Hou et al., 2018), while pepper has been shown to exhibit climacteric behavior (Paul et al., 2012; Hou et al., 2018; Dubey et al., 2019). Moreover, studies have shown that Capsicum peppers can exhibit both, making this classification inconclusive. Researchers have been studying the effects of 1-MCP on fruits and vegetables for decades, but the results are highly variable and often crop-specific. Several studies have been conducted on the use of 1-MCP in the storage and preservation of fruits and vegetables (Façanha et al., 2019; Massolo et al., 2019). The use of 1-MCP can minimize the need for low temperatures while extending shelf-life and maintaining quality. Many studies have also shown that 1-MCP treatment preserves the quality and extends the shelf-life of climacteric fruit such as “Bartlett” pear (Li et al., 2021), “Hayward” and “Qihong” kiwi (Park et al., 2014; Chai et al., 2021), strawberry (Yang et al., 2018), and tomato (Wang et al., 2016). However, no systematic, statistical analyses of the influence of different concentrations of 1-MCP on the nutritional properties, enzyme activities, and volatile compounds of “Hangjiao No.2” chili peppers during storage have been conducted. In this study, we focused on the effects of 1-MCP treatments on “Hangjiao No.2” chili peppers and specifically investigated which indicators were most affected by 1-MCP at different storage times at ambient temperature. It was also reported that 1-MCP treatment enhanced the activity of antioxidant enzymes in various plants such as broccoli florets (Yuan et al., 2010), coriander leaves (Hassan and Mahfouz, 2012), “Empire” apples (Fawbush et al., 2009), mangoes (Wang et al., 2009), and soybean plants (Djanaguiraman et al., 2011). The insignificant differences observed in MDA content between 1-MCP treatments after 3 days are in agreement with Hong et al. (2013), who discovered that 1-MCP prevented the increase in MDA content in *Psidium guajava* L. during early storage, but the effect was insignificant in the later days of storage. The activity of SOD varied among the different storage periods and 1-MCP treatments, as shown in Figure 1B. The results for SOD obtained in our study for pepper fruit are similar to the study on green asparagus exposed to 1-MCP during cold storage (Zhang et al., 2012b). The enzyme activities of POD and CAT increased before day 9 and then decreased at the end of storage regardless of the treatments, and the pepper samples treated with 0.5 μl L^–1^ 1-MCP had the highest value than the control and the treatment with 1.0 μl L^–1^. The obtained results indicate that 1-MCP treatment can significantly increase the activities of POD and CAT in pepper fruits. The 0.5 μl L^–1^ treatment was the most effective in increasing POD and CAT enzyme activities of pepper.

Vitamin C content in pepper can range from 43 mg 100 g^–1^ to 247 mg 100 g^–1^ of fresh fruits and can represent 50 to over 100% of the recommended daily intake (Wahyuni et al., 2013). The postharvest loss of nutritional values such as vitamin C can be substantial due to physical damage, relative humidity, temperature, light intensity, and prolonged storage time (Lee and Kader, 2000). Therefore, the decrease in vitamin C content observed in treated pepper fruit with increasing storage time is consistent with the fact that vitamin C, like any other antioxidant, is susceptible to degradation during the later storage period (Kalt, 2005). The highest vitamin C content in fruit treated with 1.0 μl L^–1^ and 1.5 μl L^–1^ 1-MCP compared to the control and 0.5 μl L^–1^ 1-MCP treatments during storage suggests that higher concentration of 1-MCP may have a suppressive effect on peppers (Win et al., 2006). The highest vitamin C content obtained under the 1.5 μl L^–1^ treatment during 12 days of storage was in agreement with the previous study by Jiang et al. (2018), who reported that a high concentration of 1-MCP maintained the vitamin C content of yardlong beans after 21 days of storage. According to Lee and Kader (2000), vitamin C is an antioxidant that reacts with and neutralizes singlet oxygen and other free radicals. Therefore, maintaining the vitamin C content of pepper fruits after harvest can improve fruit quality while reducing damage from reactive oxygen species. According to Barroso (2016), the average consumption of vegetables is 400 g day^–1^ per person as stated by WHO. Therefore, the health risk of a person is estimated based on the amount of vegetable consumption of a person based on 400 g day^–1^. Based on the analysis, “Hangjiao No. 2” chili peppers can be considered to be a good source of vitamin C. The acceptable daily intake of nitrate given by Gruszecka-Kosowska and Baran (2017) is 3.7 mg kg^–1^. The average nitrate levels obtained in our study in the different treatments were far below the permissible nitrate levels. This could be due to the type of fertilizer used in the production process of pepper plants (Hsu et al., 2009). The application of 1-MCP treatments inhibited the decline in protein content more than the control, especially at 1.5 μl L^–1^, which further retarded the decline in protein content at the end of storage by (34.22%) compared to the control (53.69%) (Table 1). These results support the findings that treatment with 1-MCP significantly retarded the activities associated with senescence such as protein and chlorophyll degradation in coriander leaves (Hassan and Mahfouz, 2012) and spinach leaves (Gergoff et al., 2010). The content of the total chlorophyll also decreased as storage progressed in this study. Moreover, 1-MCP at a concentration of 1.0 μl L^–1^ further retarded the degradation of chlorophyll in pepper fruit compared to the control during storage. 1-MCP has been reported to retard chlorophyll degradation in broccoli florets (Fernández-León et al., 2013), *S. melongena*, L. (Massolo et al., 2011), Yardlong beans (Jiang et al., 2018), and strawberries (Li et al., 2016). This could be the reason for the observed delay in chlorophyll content in pepper fruit treated with 1.0 μl L^–1^ during the storage period.

The aroma as a secondary metabolite is an important fruit property that is attracting the attention of a growing number of consumers and researchers. It is reported that pepper fruits contain over 200 volatile compounds (Kollmannsberger et al., 2011; Pino et al., 2011; Bogusz et al., 2012). The quality of a fruit’s aroma is determined by the type and concentration of volatiles that affects its organoleptic properties. Aromatic changes occur as a result of postharvest metabolic processes and can be influenced by a variety of pre- and postharvest factors (Sdiri et al., 2017; Fellman, 2019). The gas chromatography-mass spectrometry method was used to detect 88 volatile compounds in chili pepper “Hangjiao No.2” (Figure 2). Terpenes dominated in the pepper fruit, followed by aldehydes, esters, alkanes, alcohols, acids, phenol derivatives, ketones, and other compounds. During storage, the composition and concentration of volatile aroma compounds in the pepper fruit treated with the different treatments showed significantly different patterns with increasing storage time, especially the content of aldehydes, acids, esters, terpenes, and other aroma compounds decreased, while alcohols increased. Interestingly, alkanes showed an increasing or decreasing trend with advancing storage dates. From Figure 2, of the seven acids detected, acetic acid, oxalic acid, and hexanoic acid were the most abundant in “Hangjiao No.2” pepper fruit. Overall, the fruit treated with 1.0 μl L^–1^ treatment exhibited higher concentrations of these volatile acids than the control pepper fruit throughout the storage period. According to Wang et al. (2011b), the most important volatiles in mature fruits is aldehydes. As the second most abundant volatiles in the aroma profile, aldehydes and alcohols are known to impart the “green” notes to the aroma of fruit and are present in a wide variety of plants (Jaeger et al., 2003). The results in Figure 2 show that pepper fruit treated with 1.0 μl L^–1^ 1-MCP had a higher production of aldehydes than the fruit treated with the other treatments, suggesting that this is a consequence of the treatment. (E)-2-hexenal, which has a strong influence on the aroma of sweet peppers (Eggink et al., 2012), and Shimatogarahi (Manikharda, 2018) was also predominant in “Hangjiao No. 2” fruit. The content of (E)-2-hexenal, which exhibits fresher notes, was higher in the 1.0 μl L^–1^ treatments than in the other treatments in this study, indicating that the 1.0 μl L^–1^ treated fruit might exhibit fresher notes compared to the control. The results for aldehydes are consistent with the findings of Zhang et al. (2009), who found that the concentration of aldehydes typically decreases as the fruit ripens. Esters, the most common type of volatile, are known to impart “sweet” and “fruity” characteristics to the fruit aroma (Wang et al., 2011a). In kiwifruit, 1-MCP affected the synthesis of esters, especially ethyl butanoate and methyl butanoate, which were found to be the major esters in “Hayward” and “Qihong.” The effects of 1-MCP on volatile esters have been previously documented for apples (Lurie, 2006) and pears (Li et al., 2016). The results of Huan et al. (2020) indicated that the majority of esters increased with the ripening of kiwifruit during storage at room temperature in the control group, but 1-MCP at a concentration of 0.5 and 1.0 μl L^–1^ retarded the formation of esters in this study (Figure 2). The diverse varieties of esters in the volatile fraction of “Hangjiao No.2” was also reported in Malagueta and paprika peppers (Bogusz et al., 2012), Tabasco, Laotian, and Pebrera peppers (Rodríguez-Burruezo et al., 2010). Our results showed that all ester contents decreased or diminished under the different treatments during the storage period. A similar result was observed in apples, suggesting that the application of 1-MCP may lead to a decrease in ester production (Defilippi et al., 2005). A decrease in ester content was also observed in kiwifruit during cold storage (Günther et al., 2015). However, the treatment with 1.0 μl L^–1^ 1-MCP maintained a decrease compared to the other treatments in the present study (Figure 2). This indicates that the use of “Hangjiao No.2” treated fruit as a condiment can complement dishes due to the pleasant and fruity flavors resulting from its ester content. Terpenes are the most abundant and diverse bioactive compounds of plants in nature (Cox-Georgian et al., 2019). Terpenes are widely distributed in higher plants such as citrus, conifers, and eucalyptus and are detected in their leaves, flowers, stems, and roots (Ninkuu et al., 2021). According to Bueno et al. (2020) and Fischedick et al. (2010), terpene profile is influenced by a variety of factors such as genotypes, age of inflorescences, and environmental, production, and harvesting conditions. Although terpenes were most abundant in chili pepper “Hangjiao No.2” after harvest, their levels were relatively low compared to aldehydes in the present study, and their levels increased or diminished after treatment during the storage period, especially after day 3 of storage in the 1-MCP treatments compared to the control. 3-Carene has a pleasant aroma that translates as a rubbery odor and is common in a number of Capsicum cultivars (Oruña-Concha et al., 1998). The highest content of 3-carene, a monoterpene observed in the control, may contribute to the sweet and pungent odor with a woody character of “Hangjiao No. 2” fruit. Beta-element is one of the important terpenes produced by Jabuticabas, and it is extensively studied for its biological functions (Freitas et al., 2020). It is known for its fresh, herbaceous, and waxy aroma and ranked third among the emitted terpenes from “Hangjiao No. 2” peppers in this study. The content of terpenes was higher in the control than in the other treatments. Our results suggest that “Hangjiao No. 2” may be a natural source of β-elements, which can be used as a potential raw material for its extraction (Wang, 2012). A study by Zhu et al. (2015) revealed that nerolidol is one of the most aroma-active compounds contributing to the aroma profile of tea infusions as well as rose and apple aromas. Therefore, nerolidol could be an important contributor to the characteristic floral aroma note found in the “Hangjiao No. 2” fruit. The presence of alkanes was highlighted in Figure 2 and the relative content of alkanes showed considerable variation during the 12 days storage. Aliphatic alkanes as well as 2-methyl branched alkanes found in Shimatogarashi (Manikharda et al., 2018) and *Cupsicum frutescens* (Liu et al., 2009) domesticated in China were also present in “Hangjiao number No.2” in this study. 2-Methylpentadecane, heptadecane, 2-methyltridecane, and pentadecane were higher in “Hangjiao No. 2”. The levels of alkanes detected in this study initially increased and then declined or diminished with increasing storage time in all treated pepper fruit. The highest levels of these alkanes were detected in the treatment groups when treated with 1.0 μl L^–1^ 1-MCP. According to Rodríguez-Burruezo et al. (2010), aliphatic and methyl branched alkanes are associated with capsaicin biosynthesis, as they have been found in chili pepper species. As shown in Figure 2, “Hangjiao No.2” chili fruit started to form new alcohol on day 6 during the storage period. Beekwilder et al. (2004) stated that, the availability of alcohol, in particular, is considered as a barrier for the synthesis of esters. In this study, the 1-MCP treatments and the control significantly altered the alcohol emission of the pepper samples during the 12 days storage period. No common trend was observed for any of the detected alcohols, suggesting that these metabolites have different metabolic origins (Ortiz et al., 2010). Higher alcohol production was observed for some of the volatile compounds detected after 3 days of storage, particularly in pepper samples treated with 1.0 μl L^–1^ 1-MCP compared to 0.5 μl L^–1^ treated fruit (Figure 2), and concomitantly, with higher production of esters. A similar result was reported that 1.0 μl L^–1^ 1-MCP treatment can lead to significant changes in the alcohol production of “Tardibelle” peach fruit during storage at 20°C (Ortiz et al., 2010). The aroma compounds of Tabasco pepper include some alcohols such as 3-methyl-1-pentanol, (E)- 3-hexen-1-ol, and 1-hexanol (Azcarate and Barringer, 2010), which were also detected in “Hangjiao No. 2” fruit. 1-hexanol and 3- methyl-1-pentanol are known for their green, grassy, fresh aroma (Azcarate and Barringer, 2010; Zhu et al., 2015). This suggests that the highest concentration of these compounds detected in 1.0 μl L^–1^ of the treated “Hangjiao No. 2” fruit (Figure 2) is likely an important contributor to flavor. Bogusz et al. (2012) reported that α-ionone and β-ionone were detected as degradation products of carotenoids in Brazilian chili peppers with low aroma threshold and characterized as fruity or floral. However, only β-ionone was detected in the pepper “Hangjiao No.2” after harvest, and it decreased in all treatments during the storage period. According to Zimmermann and Schieberle (2000), β-ionone plays a significant role as an odor-active component in pepper odor, which is still developing at all three stages. This implies that the highest β-ionone content observed in the control treated fruit may contribute a fruity or floral aroma to “Hangjiao No.2”. The group of phenolic derivatives was identified by the presence of methyl salicylate, a compound found mainly in unripe *S. lycopersicum* L. (Lewinsohn et al., 2005). Although methyl salicylate was detected in “Hangjiao No.2” pepper fruit in all treatments throughout the storage period, its characteristic sweet aroma decreases with increasing storage time. However, the highest levels of this phenol ester were found in the 0.5 and 1.5 μl L^–1^ 1-MCP treated fruit at the end of storage. This means that the fruit treated with 0.5 and 1.5 μl L^–1^ 1-MCP could have a more pronounced sweet-green mixed aroma than the control and the treatments with 1.0 μl L^–1^ 1-MCP. In addition, 6 other volatiles were detected in the fruit of chili pepper “Hangjiao No.2” after harvest, including methyl benzhydroximate, 2-pentyl-thiophene, dihydro-5-pentyl-2(3H)-furanone, 2-methoxy-3-(1-methylpropyl)-pyrazine, m-xylene, and 2-methoxy-3-(2-methylpropyl)-pyrazine. Among these compounds, methyl benzhydroximate was the most abundant, followed by 2-methoxy-3-(2-methylpropyl)-pyrazine and m-xylene while 2-pentylthiophene, dihydro-5-pentyl-2(3H)-furanone and 2-methoxy-3-(1-methylpropyl)-pyrazine were present in small amounts. Most compounds belonging to this group were relatively stable on day 9 of storage, but decreased dramatically or disappeared after day 6 of storage. In addition, methyl benzhydroximate and 2-methoxy-3-(2-methylpropyl)-pyrazine appeared in 1.0 and 1.5 μl L^–1^ of pepper fruit treated with 1-MCP throughout the storage period, but treatment with 0.5 μl L^–1^ 1-MCP inhibited the production of methyl benzhydroximate after treatment. Mazida et al. (2005) and Pino et al. (2006) found that methoxypyrazines are the major flavor components in green peppers and are responsible for its distinctive green aroma. Analysis of volatile aroma compounds of fresh chili (*C. annuum*) at different stages of maturity using solid phase microextraction (SPME) have also shown that methoxypyrazines, especially 2-methoxy-3-(2-methylpropyl) pyrazine, have a stronger influence on the quality and odor intensity of many varieties of *C. annum*, *baccatum*, and *pubescens* (Rodríguez-Burruezo et al., 2010). According to Mazida et al. (2005), 2-methoxy-3-(2-methylpropyl) pyrazine and hexanal decreased with fruit ripening during storage, and the color also changed. These results suggest that the further delay in the decrease of 2-methoxy-3-(2-methylpropyl) pyrazine observed in pepper fruit treated with 1.0 and 1.5 μl L^–1^ 1-MCP may be due to the fact that the treatments with 1-MCP at 1.0 and 1.5 μl L^–1^ suppressed the degreening process with increasing storage time.

## Conclusion

In this study, the positive effects of 1-MCP treatments on the nutritional properties of pepper fruit during storage at ambient temperature were found. We demonstrated that the application of 1-MCP inhibited the increase of nitrate content and delayed the degradation of chlorophyll, vitamin C, and protein of “Hangjiao No.2” peppers. In addition, 1-MCP had the greatest effect on the activities of antioxidant enzymes such as SOD, POD, and CAT and decreased the accumulation of MDA in pepper fruit. However, 1-MCP treatments did not have any effect on the shelf-life of this pepper cultivar during the 12 days storage period. The study also revealed that terpenes dominated the “Hangjiao No.2” chili fruit, followed by aldehydes, esters, alcohols, alkanes, acids, phenol derivatives, ketones, and other volatile compounds. During storage, significant changes were observed in the pepper samples treated with the different treatments with increasing storage time. The desired volatile compounds including aldehydes, esters, alkanes, and alcohol classes in the “Hangjiao No.2” chili fruit treated with 1.0 μl L^–1^ 1-MCP showed higher retention during the storage period as compared to the other treatments applied. 1-MCP at a concentration of 1.0 μl L^–1^ was found to be the most suitable concentration of all the applied treatments. This study shows that the application of different treatments and storage times can affect the aroma composition, nutritional properties, and enzyme activities and provide important information about “Hangjiao No.2” that could be useful in promoting pepper fruit for future marketing and processing.

## Data Availability Statement

The raw data supporting the conclusions of this article will be made available by the authors, without undue reservation.

## Author Contributions

EB and JZ: conceptualization. KH, YM, and YC: methodology. EB: software, formal analysis, investigation, data curation, writing—original draft preparation, and writing—review and editing. JX and JZ: validation, visualization, and supervision. JX: resources, project administration, and funding acquisition. All authors have read and agreed to the published version of the manuscript.

## Conflict of Interest

The authors declare that the research was conducted in the absence of any commercial or financial relationships that could be construed as a potential conflict of interest.

## Publisher’s Note

All claims expressed in this article are solely those of the authors and do not necessarily represent those of their affiliated organizations, or those of the publisher, the editors and the reviewers. Any product that may be evaluated in this article, or claim that may be made by its manufacturer, is not guaranteed or endorsed by the publisher.
