# Supplementary material for: Storage Stability of Nutritional Qualities, Enzyme Activities, and Volatile Compounds of “Hangjiao No. 2” Chili Pepper Treated With Different Concentrations of 1-Methyl Cyclopropene
Source: Front Plant Sci. 2022 Mar 8;13:838916. doi: 10.3389/fpls.2022.838916 (PMC8957985; doi:10.3389/fpls.2022.838916)
Supplement: Supplementary file 1 [file Table_1.DOCX]

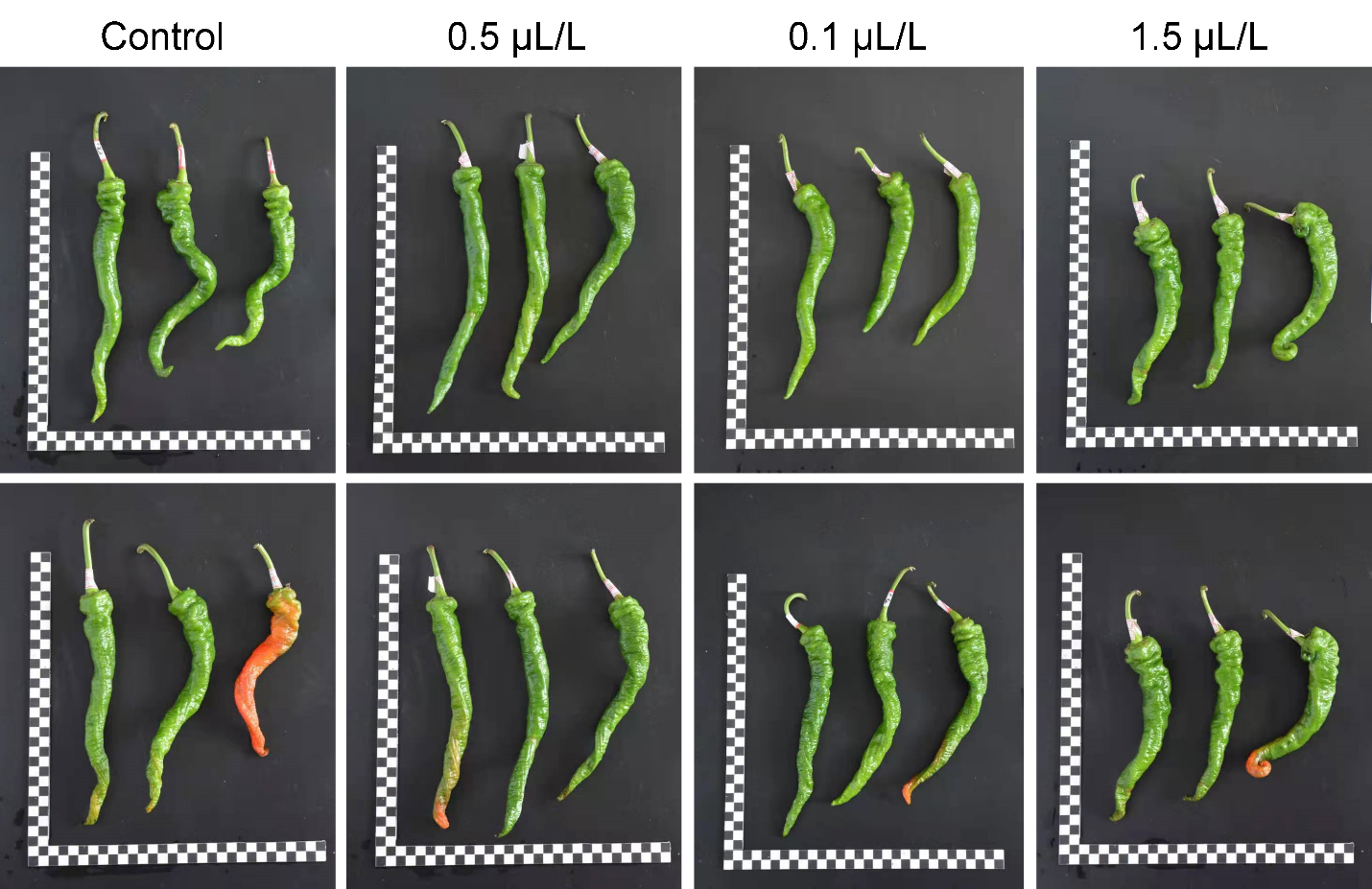


Appearance of fruits of 'Hangjiao No. 2' chili pepper at ambient temperature during storage in the control and different concentrations of 1-MCP treatment.
